# Development of expression vectors for *Escherichia coli *based on the pCR2 replicon

**DOI:** 10.1186/1475-2859-6-14

**Published:** 2007-05-10

**Authors:** Rupali Walia, J K Deb, K J Mukherjee

**Affiliations:** 1 Department of Biochemical Engineering and Biotechnology, Indian Institute of Technology Delhi, New Delhi, India; 2 School of Biotechnology, Jawaharlal Nehru University, New Delhi, India

## Abstract

**Background:**

Recent developments in metabolic engineering and the need for expanded compatibility required for co-expression studies, underscore the importance of developing new plasmid vectors with properties such as stability and compatibility.

**Results:**

We utilized the pCR2 replicon of *Corynebacterium renale*, which harbours multiple plasmids, for constructing a range of expression vectors. Different antibiotic-resistance markers were introduced and the vectors were found to be 100% stable over a large number of generations in the absence of selection pressure. Compatibility of this plasmid was studied with different *Escherichia coli *plasmid replicons viz. pMB1 and p15A. It was observed that pCR2 was able to coexist with these *E.coli *plasmids for 60 generations in the absence of selection pressure. Soluble intracellular production was checked by expressing GFP under the *lac *promoter in an expression plasmid pCR2GFP. Also high level production of human IFNγ was obtained by cloning the h-IFNγ under a T7 promoter in the expression plasmid pCR2-IFNγ and using a dual plasmid heat shock system for expression. Repeated sub-culturing in the absence of selection pressure for six days did not lead to any fall in the production levels post induction, for both GFP and h-IFNγ, demonstrating that pCR2 is a useful plasmid in terms of stability and compatibility.

**Conclusion:**

We have constructed a series of expression vectors based on the pCR2 replicon and demonstrated its high stability and sustained expression capacity, in the absence of selection pressure which will make it an efficient tool for metabolic engineering and co-expression studies, as well as for scale up of expression.

## Background

Using techniques of metabolic engineering *E.coli *has grown from a host used for the production of simple proteins, to a sophisticated platform which can be utilized to express complex proteins in their biologically active form and also complete metabolic pathways which require a calibrated and simultaneous expression of multiple genes. There is thus a need to develop and design versatile vectors for *E.coli *to serve as tools for the above purpose. Improvement of vectors are being carried out so that they can be used for special purposes, such as for the synthesis of proteins that can be easily recovered and purified [1], for directing the protein synthesized to specific compartments and thus facilitate proper folding [2] and also secretion to the extracellular medium [3]. Many proteins need to be produced in large quantities, especially those that have commercial significance, like industrial enzymes or therapeutic proteins. In such cases issues of plasmid stability [4] are critical for getting sustained production during the scale up of cultures to industrial levels [5].

The use of two plasmid systems where the gene of interest is expressed by the first plasmid and the second plasmid contains genes which aid this expression, has found wide usage. Such a two plasmid system requires a set of vectors with different origins of replication, which are not only compatible but also able to co-exist stably over a large number of generations [6]. Notable examples are expressing the T7 RNA polymerase gene in a second plasmid to drive the over-expression of the gene downstream of the T7 promoter [7], co-expression of different enzymes for pesticide degradation [8], the co-expression of chaperons like GroEL-GroES to help in the proper folding of the expressed protein [9], expression of genes involved in glycosylation [10] or other post translational modifications like tyrosine sulfation [11] in *E.coli*. The ability to clone and express all genes in a complete pathway, allows us to metabolically engineer cells to produce novel metabolites or enhance production levels of metabolites [12,13]. These developments underscore the need to discover and construct new plasmid vectors, especially those that are compatible with other *E.coli *plasmids.

There are several reports describing the construction of novel and useful vectors with properties such as stability and compatibility with existing plasmids [14,15]. However most of these reports do not demonstrate sustained expression abilities and long term co-existence, especially during high level production of recombinant proteins, when the cell is under a severe metabolic burden. To this end, we have used a new pCR2 replicon for the construction of expression vectors which have high level stability and sustained expression capability in the absence of selection pressure.

## Results and discussion

### Characterisation of pCR2 plasmid

**Figure 1:**
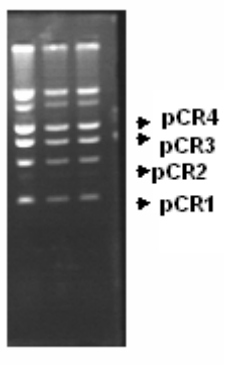
Plasmid profile of *C. renale *showing the presence of multiple plasmids.pCR1 (1.4 kb), pCR2 (3.2 kb), pCR3(4.4 kb), pCR4 (5.7 kb).

**Figure 2:**
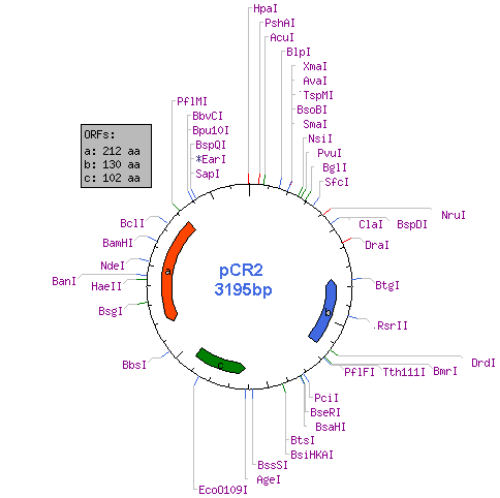
Plasmid map of pCR2 showing useful restriction sites and three ORFs.

Total plasmid DNA (Figure 1) was isolated from an overnight culture of *Corynebacterium renale *which showed the presence of multiple bands. These bands correspond to four distinct plasmids viz pCR1, pCR2, pCR3 and pCR4 which has been demonstrated earlier by restriction analysis and southern hybridisation [16,17]. Of these, the 3.2 kb plasmid pCR2 was gel eluted, digested with *Hinc*II to generate two fragments which were separately cloned onto a pUC19 vector. Sequencing of these inserts was done by primer walking, allowing us to obtain the complete nucleotide sequence of the 3.2 kb pCR2 plasmid which has been annotated and deposited in GENBANK (Accession No. EF488047). The plasmid had a GC content of 52%. The analysis for open reading frames showed the presence of three distinct ORFs (Figure 2). A putative region involved in multimer resolution, similar to pEC029 *E.coli *plasmid was also found. A complete restriction map was prepared to identify the restriction sites suitable for inserting different antibiotic resistance markers and expression cassettes into the pCR2 backbone. Plasmid copy number was determined by gel densitometric scanning [18] and found to vary between 25–35.

### Construction of pCR2amp and pCR2cat and stability studies

*E.coli *plasmid pET22b was taken as the source of the ampicillin cassette. Suitable primers were designed with *Cla*I restriction enzyme sites at both ends. The PCR amplicon of 1.1 kb was digested with *Cla*I and introduced into the *Cla*I site of pCR2 to get pCR2amp. The construct so obtained had a size of 4.3 kb. Similarly, the *E.coli *plasmid pACYC184 was taken as the source of the chloramphenicol cassette. Plasmid pACYC184 was digested with *Acc*I and *BstB*I, to release the complete chloramphenicol cassette of 1.1 kb and ligated to pCR2 digested with *Cla*I. The recombinant plasmid so obtained had a size of 4.3 kb and was designated as pCR2cat. The clones were confirmed by sequencing and used as a basic backbone for the construction of different expression vectors. As a first step, the stability of pCR2amp and pCR2cat was studied by repeated sub-culturing for six days, as described in materials and methods. Both the plasmids were found to be 100% stable for 60 generations demonstrating the extremely high level stability of this replicon in *E. coli*.

### Compatibility studies of the pCR2 replicon with other *E.coli *plasmids

Even though many plasmid vectors exist in *E.coli*, these are mostly derived from the same parent plasmid and hence the range of distinct (and therefore compatible) origins is limited. Thus, most of the commonly used commercially available vectors have either pMB1(pUC, pET, pRSET series), p15A (pACYC184, pGP1-2) or ColE1 (pBacTag series) origins of replication. Of these, p15A based plasmids [19] are known to be unstable over a large number of generations in the absence of selection pressure.

**Figure 3:**
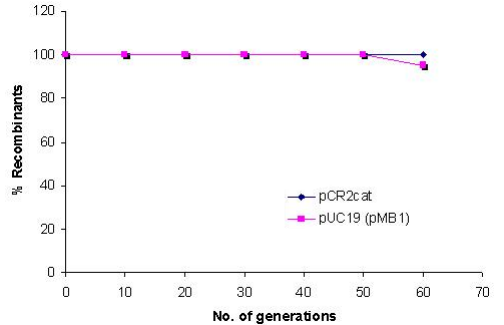
Stability and compatibility of pCR2 replicon with pMB1 replicon (pUC19): *E.coli *DH5α cells co-transformed with pCR2cat and pUC19 were grown in LB with repeated sub-culturing in the absence of antibiotics.

**Figure 4:**
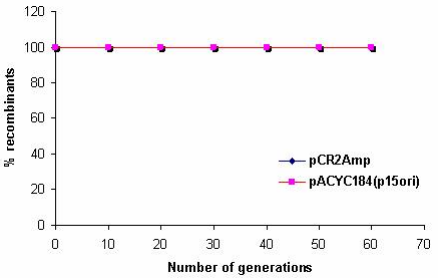
Stability and compatibility of pCR2 replicon with p15 replicon (pACYC184): *E.coli *DH5α cells co-transformed with pCRamp and pACYC184 were grown in LB with repeated sub-culturing in the presence of chloramphenicol to ensure the existence of pACYC184.

The stability and compatibility of the pCR2 replicon was tested with pUC19 and pACYC184. For this *E.coli *DH5α cells were cotransformed with pUC19 and pCR2cat. The coexistence of both plasmids was checked by repeated sub-culturing in the absence of antibiotics. pCR2cat was found to be compatible with pUC19 and stable coexistence was observed for 60 generations (Fig 3). This demonstrated that the stability of the pCR2 replicon was not affected by the presence of a second plasmid. Similarly *E.coli *DH5α cells were cotransformed with pCR2amp and pACYC184. However since pACYC184 is known to be an unstable plasmid, its presence was ensured by adding chloramphenicol in the culture medium while ampicillin was not added. The coexistence of the two replicons was checked for six days (~60 generations) and they were found to coexist stably for several generations, even in the absence of selection pressure for pCR2 (Fig 4). The above experiments demonstrated the stability and suitability of the pCR2 plasmid for co-expression studies.

### Construction of pCR2 expression vector (pCR2-*P*_*lac*_-GFP) having *P*_*lac *_promoter and GFP as the reporter gene

Plasmid pNER41 containing a GFP reporter gene under the *lac *promoter was digested with *Pst*I and *Nsi*I to release a 2.2 kb fragment containing the GFP gene along with the *lac *promoter and a transcription terminator. This 2.2 kb fragment was ligated to pCR2amp digested with *Nsi*I resulting in the recombinant plasmid pCR2-*P*_*lac*_-GFP. The screening of positive clones was based on the fluorescence of transformed colonies under IPTG induction. The clones were further confirmed by restriction analysis and sequencing.

### Shake flask studies on the stability of expression of pCR2-*P*_*lac*_-GFP in *E.coli*

**Figure 5:**
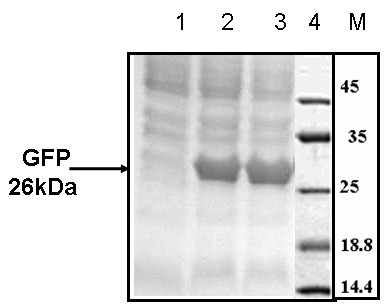
Expression of GFP in *E.coli *DH5α containing pCR2-*P*_*lac*_-GFP. Total cell lysates run on 15% SDS-PAGE at different time points post induction. Lane 1: 0 hr. Lane 2: 4 hrs Lane3: 6 hrs. Lane 4: Protein molecular weight marker.

The recombinant cells were grown to mid exponential phase (OD_600_- 0.4 to 0.6) in shake flasks, in LB containing ampicillin, induced by IPTG and the level of protein expression checked by SDS-PAGE. An induction in the form of a 26 kDa protein band, which is the expected molecular weight of the protein, was observed post induction (Fig 5). The protein was produced in a soluble form, in the cytoplasm and the high level of fluorescence indicated that the protein was expressed in its biologically active form.

**Figure 6:**
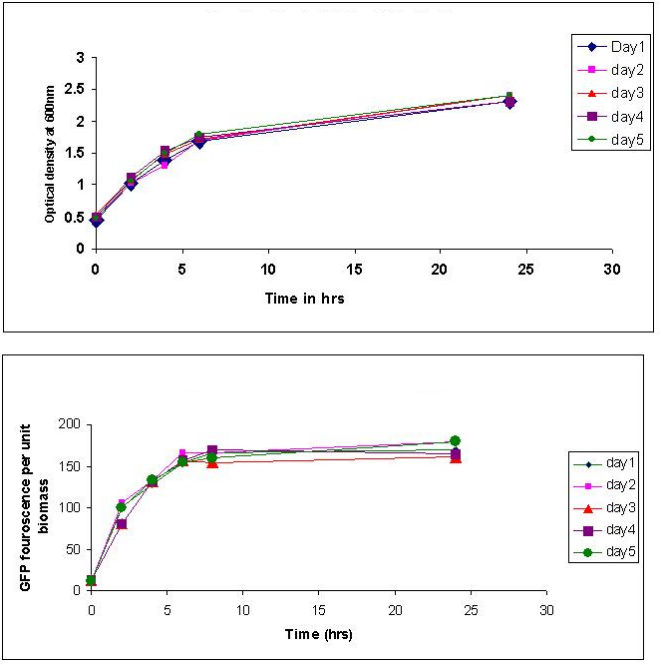
Growth and expression profile of pCR2-*P*_*lac *_-GFP recombinant cells. *E.coli *DH5α cells transformed with pCR2-*P*_*lac *_-GFP were grown in shake flask with sub-culturing in the absence of selection pressure for 5 days. a) Growth profile b) Fluorescence per unit biomass.

To check the sustainability of expression over a large number of generations, the recombinant cells were grown in the absence of selection pressure, with repeated sub-culturing as described in materials and methods. Two flasks were used everyday, in which one of the flasks was induced at mid exponential phase to study the production profile, and the uninduced flask was used for sub-culturing after 24 hours of growth. It was observed that the production levels as measured by culture fluorescence increased rapidly for the first six hours post induction after which it gradually tapered off. From the biomass profile it was clear that the slight increase in culture fluorescence after six hours post induction was primarily due to the equivalent increase in biomass concentration. Thus the GFP activity per unit biomass tended to plateau after 6 hrs of induction which possibly represents the upper limit of the fraction of soluble recombinant protein inside the cell. This production pattern was checked over 6 days with repeated sub-culturing and no significant change was observed (Figure 6a, 6b) demonstrating not only high stability but also sustained expression capability. Since P_*lac *_is not a strong promoter, the expression level is dependent on the number of copies of the GFP gene present inside the cell. Copy number was measured and no significant fall was observed during the six days of sub-culturing. Thus the results also indicate the maintenance of adequate copy number during sub-culturing in order to get sustained expression levels at a constant value. This study demonstrates the usefulness of this expression vector in scale up studies.

### Construction of a T7 promoter based pCR2 expression vector (pCR2-IFNγ)

The T7 expression system is routinely used for getting high-level production of recombinant proteins in *E. coli*. We chose a previously constructed recombinant clone pRSET-IFNγ [20] in which the human interferon gamma gene was cloned in the MCS of pRSET-A. Suitable primers were designed with *Nsi*I restriction sites at the ends, to amplify the complete expression cassette from the pRSET-IFNγ clone (consisting of the T7 promoter, the h-IFNγ gene in the MCS along with the transcriptional terminator). The PCR amplified fragment was digested with *Nsi*I and cloned at the *Nsi*I site of the pCR2amp plasmid. The resultant clones were confirmed by restriction analysis and PCR amplification (using the same gene specific primers), followed by sequencing. The vector so constructed was designated pCR2-IFNγ and had a size of 5 kb.

### Expression studies with recombinant pCR2-IFNγ

**Figure 7:**
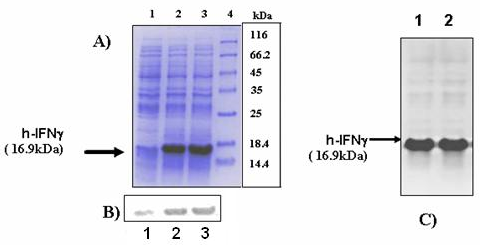
Expression of h-IFNγ in *E.coli *BL21 (DE3) containing pCR2-IFNγ. a) Total cell lysates run on 15% SDS-PAGE at different time points post induction. Lane 1: Pre induction sample. Lane 2: 4 hrs sample. Lane 3: 6 hrs sample. Lane 4: Protein molecular weight marker. (b) Western blot profiles showing expression of pCR2-IFNγ at the above time points. (c) Comparative expression of h-IFNγ in *E.coli *BL21 (DE3) cells vis-a-vis expression in *E.coli *DH5α heat shock system. Total cell lysate run on 15% SDS-PAGE 4 hrs post induction Lane 1: IPTG induced *E.coli *BL21 (DE3) containing pCR2-IFNγ. Lane 2: Temperature induced *E.coli *DH5α containing pCR2-IFNγ and pGP1-2.

Interferon gamma expression was initially checked by transforming *E. coli *BL21 (DE3) cells by pCR2-IFNγ. These host cells which contain the T7 RNA polymerase gene in the chromosome under the *lac *promoter. The transformed cells were grown in LB containing ampicillin to mid exponential phase (OD_600 _0.4–0.6), induced by IPTG and the level of protein production was checked in the post induction samples by SDS-PAGE. A protein band was observed post induction at 16.9 kDa, which is the expected molecular weight of the protein. The level of expression was very high and IFNγ constituted ~30% of the total cellular protein after 4 hrs of induction (Figure 7a). Total cell lysates were prepared by sonication and centrifuged. The IFNγ band was obtained in the pellet demonstrating that it was primarily produced as inclusion bodies. Western blot was used to confirm the expression of IFNγ (Figure 7b).

To assess the long term compatibility of pCR2-IFNγ with another *E.coli *plasmid it was decided to try an alternate expression strategy where multiple copies of T7 RNA polymerase gene are present on a second plasmid (pGP1-2) under the heat inducible λP_L _promoter [7]. *E. coli *DH5α was cotransformed with pCR2-IFNγ and pGP1-2 and the cells harboring the two plasmids were grown in the presence of ampicillin and kanamycin to mid exponential phase (OD_600 _– 0.4 to 0.6) at 30°C to repress expression. The culture was induced by heat shock at 42°C for 5 min and expression was checked post induction. From the SDS-PAGE it was clear that the IFNγ levels obtained by both expression systems i.e IPTG vs heat induction were nearly equal (Figure 7c). It is well known that T7 RNA polymerase production levels are higher in the dual plasmid system utilizing heat shock. However the fact that this did not lead to a correspondingly higher h-IFNγ in the heat induced system, demonstrates that the transcription step was not the rate limiting step in IFNγ expression. This is similar to earlier observations by researchers, who have shown that the rate limiting step in a T7 expression system is usually translation initiation [21].

**Figure 8:**
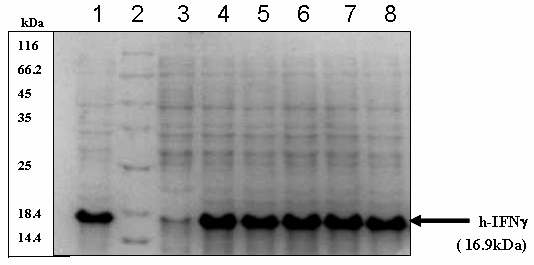
Expression of h-IFNγ over repeated rounds of subculturing in the absence of selection pressure. *E.coli *DH5α transformed with pCR2-IFNγ and pGP1-2 were grown without ampicillin at 30°C with subculturing for six days. Total cell lysates run on 15% SDS-PAGE. Lane 1: 4^th ^hr post induction sample from Day 1. Lane 2: Protein molecular weight marker. Lane 3: Uninduced culture Lane 4–8: 4^th ^hr post induction samples from day 2 to day 6.

To check the sustainability of expression of this system it was decided to subculture *E.coli *DH5α cells containing both the plasmids, without selection pressure for pCR2-IFNγ. However to ensure the presence of pGP1-2, in subsequent rounds of subculturing it was decided to maintain the selection pressure on this plasmid by adding kanamycin to the culture medium. An overnight culture of recombinant cells, containing both the plasmids, was used to inoculate two flasks of 100 ml LB without ampicillin. While one of the flasks was induced by heat shock, the second flask was not induced and allowed to grow for 24 hrs at 30°C thus representing 10 generations of uninduced growth (as described in material and methods). The above procedure was repeated for six days and the production levels were monitored daily, allowing correspondingly larger numbers of generations to elapse before inducing the culture. As in the case of GFP, a similar expression pattern was obtained and there was no decrease in the production levels of h-IFNγ (Figure 8).

This study conclusively demonstrated the stability and the sustained high level expression capacity of the pCR2-IFNγ construct, in the absence of selection pressure.

## Conclusion

We have constructed expression vectors utilizing the pCR2 replicon and shown sustained expression of two model proteins viz GFP and IFNγ under different promoters. The pCR2 replicon is highly stable and compatible with a range of *E.coli *vectors. This property of pCR2 has potential commercial utility since it can be used for high level production of proteins in large scale fermenters, even in the absence of antibiotics.

## Materials and methods

### Bacterial strains and plasmids

*E.coli *DH5α and BL21 (DE3) strains were obtained from Novagen. *Corynebacterium renale *was grown at 37°C in Luria broth. *E. coli *plasmids pUC19 and pACYC184 were obtained from New England Biolabs. pET22b was obtained from Novagen. pRSET-IFNγ was constructed by cloning the h-IFNγ gene in a commercially available vector pRSETA [20]. For this total RNA was isolated from H37RV induced human PBMCs, suitable primers having *Nde*I and *BamH*I restriction sites were designed to amplify the IFNγ-cDNA by RT-PCR and the amplified h-IFNγ gene was cloned into the commercially available plasmid pRSET-A (Invitrogen). pNER41 was a generous gift from John Archer, Department of Genetics, Cambridge UK. It has a kanamycin resistance gene, a GFP*mut3 *reporter gene under the *P*_*lac *_promoter. The mut3 phenotype allows GFP to be expressed primarily as a soluble protein. The plasmid pGP1-2 has a kanamycin resistance gene, the origin of replication was taken from pACYC177 and it is used for the expression of the T7 RNA polymerase gene under the heat inducible λP_L _promoter [22].

### DNA manipulations

Plasmid DNA from *Corynebacterium renale *was isolated by a modification of the alkaline lysis method [23] where the cells were initially incubated at 37°C for 2 hrs with 2 mg/ml lysozyme in Tris- glucose EDTA buffer. Plasmid DNA from *E.coli *was isolated by alkaline lysis method according to the standard procedure [23]. Transformation was performed using an electroporator (Biorad Micropulser, USA). For this cells were grown in 100 ml LB to an OD_600 _of 0.6, chilled on ice and harvested by centrifugation (10 min, 4000 g at 4°C). The pellet was washed twice with 100 ml of distilled water and once with 10% glycerol. The washed pellet was resuspended in 5 ml of distilled water. Aliquots of 40 μl were mixed with 15 ng of plasmid DNA in chilled electroporation cuvettes (0.1 cm electrode gap). A pulse of 1.8 kV was applied and 1 ml SOC was immediately added. The culture was then revived for 2 hrs at 37°C before plating on antibiotic plates.

Plasmid copy number was determined according to the method of Seelke et.al [18]. Typically plasmid DNA was extracted by alkaline lysis method from equal cell masses of overnight or exponentially growing cultures in antibiotic supplemented Luria broth. Equal amounts of plasmid- containing extract were then electrophoresed in agarose gels containing EtBr and plasmid bands were visualised under UV and quantitated by BIORAD Quantity One software.

Restriction enzymes, T4 DNA ligase and other DNA modifying enzymes purchased were from MBI Fermentas (GmbH) and New England Biolabs (USA). Cloning procedures, agarose gel electrophoresis and SDS-PAGE were performed according to standard procedures [23]. DNA fragments were isolated from agarose gels by using the QIAquick gel extraction kit (QIAGEN). Primers were synthesised from Microsynth (Switzerland).

The primers used for amplification of ampicillin cassette were

(Forward primer) ampF: 5' CGT TT**A TCG AT**T CAG GTG GCA CTT TTC GG 3'

(Backward Primer) ampR: 5' CTT TT**A TCG AT**G GTC TGA CGC TCA GTG GA 3'

The underlined sequence represents *Cla*I recognition site.

The sequence of primers used for amplifying the T7-IFNγ expression cassette

Forward primer F: 5 'GAT **ATG CAT**CCC GCG AAA TTA AT 3'

Reverse primer R : 5' TAG AGG CCC CAA **ATG CAT**ATG CTA G 3'

The underlined sequence represents *Nsi*I recognition site.

### Stability and compatibility studies

For studying stability a culture of *E.coli *DH5α cells containing the plasmid were grown in LB medium containing the appropriate antibiotic at 37°C from a single colony. An aliquot of 100 μl of this overnight culture was inoculated in 100 ml of fresh LB medium without antibiotic and grown for 24 hrs till the cells reached stationary phase. At this point the OD_600 _of the culture was typically between 2.0–2.4. The cfu/ml was calculated by counting the total number of colonies obtained after dilution plating which was found in the range of ~2 × 10^9 ^cells/ml. The fraction of non recombinants emerging in the culture was calculated by plating appropriate dilutions of this culture onto LB plates (without antibiotics) to get isolated colonies. More than 50 colonies were transferred onto LB antibiotic plates and the fraction of plasmid containing cells was calculated by counting the number of colonies which grew on the antibiotic plate. An aliquot (100 μl) of this stationary phase culture was used to inoculate 100 ml of fresh medium and this process of sub-culturing was repeated for six days. Since each inoculum was 0.1% (100 μl in 100 ml) it represented a 1000 fold increase in the cell number. This was also because both the OD and cfu/ml values were similar at the end of each sub-culturing. Thus every 24 hr period of growth would represent 10 doublings and hence 10 generations. For compatibility studies *E.coli *DH5α cells were co-transformed with two plasmids and the stability of both the plasmids was tested independently.

### Expression of pCR2-IFNγ in shake flasks

For IPTG induced system *E.coli *BL21 (DE3) competent cells were transformed with pCR2-IFNγ. Primary culture was grown at 37°C at 200 rpm in 10 ml LB containing 100 μg/ml ampicillin. This culture was used to inoculate 100 ml of LB with antibiotic, allowed to grow till mid log phase (OD_600 _– 0.4 to 0.6) and induced by 1 mM IPTG. Samples were collected at 2 hour intervals for 8 hrs and analyzed on SDS-PAGE. For heat induced dual plasmid system *E. coli *DH5α cells were cotransformed with pCR2-IFNγ and pGP1-2 The cells harboring both the plasmids were grown in LB containing ampicillin (100 μg/ml) and kanamycin (50 μg/ml) to mid exponential phase at 30°C (thereby repressing the λP_L _promoter). The culture was induced by heat shock in a shaking water bath at 42°C for 5 min. The flask was then shifted to 37°C for further growth. Samples were collected at 2 hour intervals for 8 hrs and analyzed on SDS -PAGE. The stained gels were scanned using a Gel-Documentation system (Gel Doc™, Biorad) and analyzed using Quantity One Software (Biorad).

### Western Blot

The proteins were separated on a 12.5% SDS-PAGE and transblotted onto a nitrocellulose (NC) membrane in a buffer containing 25 mM Tris HCl pH 8.3, 192 mM glycine and 20% methanol. After the transfer, the NC membranes were incubated in PBST (10 mM) containing 2% BSA for 90 minutes to block additional protein binding sites. After a brief wash with PBST, the membrane was incubated with anti-hIFNγ polyclonal antibodies (rabbit IgG, 1:10,000 dilution) for 1 hour at room temperature with gentle rocking. The membrane was then washed 3 times for 10 minutes in PBST and incubated in anti-rabbit IgG conjugated to Horse Radish Peroxidase (HRPO; 1:10,000 dilution)/alkaline phosphatase (AP; 1:10,000 dilution) at RT for 1 hours with gentle rocking. Finally, the membrane was washed 3 times for 10 minutes in PBST and the immunoreactive bands were visualized by 9 mg DAB and 20 μl H_2_O_2 _solution in 10 ml of 100 mM Tris HCl, pH 7.6, until the bands develop to the desired intensity.

### Sustained expression capacity

To check the sustainability of expression over a large number of generations, the recombinant cells were grown in the absence of selection pressure with repeated sub-culturing as described previously in the segregation stability tests. Two flasks were used everyday, in which one of the flasks was induced at mid log phase, to study the production profile and the uninduced flask was used for sub-culturing after 24 hours of growth.

### a) Sustained expression capacity of pCR2-IFNγ in heat inducible dual plasmid system

*E. coli *DH5α recombinant cells harbouring plasmids pCR2-IFNγ and pGP1-2 were grown overnight in LB containing ampicillin and kanamycin at 30°C and 200 rpm. 100 μl of this overnight culture was used to inoculate two flasks of 100 ml LB containing only kanamycin (50 μg/ml) to ensure the presence of pGP1-2 in the subsequent generations while eliminating the selection pressure on pCR2-IFNγ. One of the flasks was induced by heat shock (at OD_600 _0.4–0.6), transferred to 37°C and samples collected every two hours post induction for 8 hours. These samples were run on 12% SDS PAGE to check the level of IFNγ expression. The second flask was not induced and allowed to grow at 30°C for 24 hr thus representing 10 generations of uninduced growth. Sub-culturing was done by inoculating two flasks of LB (100 ml) as described previously. The expression levels were monitored consecutively for six days.

### b) Sustained expression capacity of pCR2-*P*_*lac*_-GFP

*E. coli *DH5α cells harbouring pCR2-*P*_*lac*_-GFP plasmid were grown overnight in LB containing ampicillin at 37°C, 200 rpm. 100 μl of this overnight culture was used to inoculate two flasks of 100 ml LB without antibiotic. One of the flasks was induced by 1 mM IPTG when the cells were in mid exponential phase (OD_600 _0.4–0.6) and samples were collected every two hours post induction for 8 hrs. The bioactivity of GFP was measured by fluorescence by exciting the samples at 490 nm and reading the emission at 514 nm in a Cary Eclipse Fluorescence Spectrophotometer (Varian, Netherlands). The second flask was not induced and allowed to grow for 24 hr thus representing 10 generations of uninduced growth and used for subculturing. The production levels were monitored consecutively for six days.

## Abbreviations

GFP Green fluorescent protein

IPTG Isopropyl-β-D-galactoside

LB Luria Bertani

PCR Polymerase chain reaction

SDS-PAGE Sodium dodecyl sulphate- polyacrylamide gel electrophoresis.

ORFs Open reading frame

MCS Multiple cloning site

PBST Phosphate buffered saline tween

HRP Horse Radish Peroxidase

DAB 3,3' Diaminobenzidine

RT Room temperature

IBs Inclusion bodies

EtBr Ethidium bromide

## Authors' contributions

RW is a PhD scholar who worked under the supervision of JKD and KJM.
